# William John Little (1810–1894)

**DOI:** 10.1007/s00415-015-7890-5

**Published:** 2015-09-04

**Authors:** Krzysztof Pietrzak, Andrzej Grzybowski, Jacek Kaczmarczyk

**Affiliations:** Department of Orthopaedics and Traumatology, University of Medical Sciences, Poznan, Poland; Department of Ophthalmology, Poznań City Hospital, Poznan, Poland; Department of Ophthalmology, University of Warmia and Mazury, Olsztyn, Poland

**Fig. 1:**
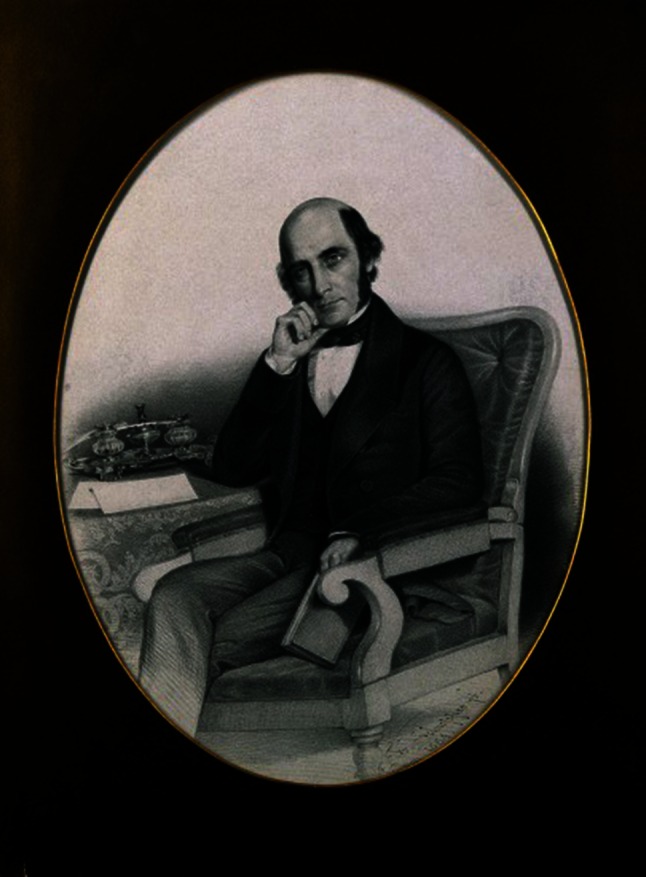
William John Little, 1854 (image reproducer with permission from the Wellcome Library, London)

William John Little (Fig. 1) was born on 7 August 1810 in London, where his father owned an inn. Little had suffered from club foot from early childhood. It is still unclear whether it was a birth defect or an aftermath of an early childhood polio. Having completed his education in England, he continued his studies in France. His language ability was outstanding; in French language contests he came ahead of native Frenchmen.

He returned to London in 1826 to start a 2-year practice in a pharmacy. In 1828 he started medical studies at the London Hospital, and later continued his medical education at Guy’s Hospital. Soon, he became a teacher of anatomy, physiology and pathology at the London Hospital. In 1832, Little was admitted to the Royal College of Surgeons. However, his attempts to obtain a post as surgeon at the London Hospital were unsuccessful, which led him to move to Berlin in 1834 to pursue further education.

During his studies in Europe he met Luis Stromeyer (1804–1876), a pioneer of tenotomy. Stromeyer performed a very successful tenotomy of the Achilles tendon on Little’s foot. Little was impressed both by Stromeyer’s achievements and his own experience as a patient. His work [1] on the treatment of foot deformities earned him a doctoral degree from the University of Berlin in 1837.

Little returned to London in 1837. In the same year, he performed a tenotomy of Achilles tendon on a 15-year-old boy. He developed his private practice and in 1839 he published a paper on club foot treatment [2]. The surgeries were very successful, which made Little more and more recognized, both as a practitioner and a theorist. In 1840 he received a much desired job as surgeon at the London Hospital. In the same year, on Bloomsbury Square, he opened the world’s first hospital dedicated solely to treating orthopaedic disorders, which later came to be known as the Royal Orthopaedic Hospital of London.

His experience, both as a patient and a doctor, in particular in treating young children, resulted in a series of papers in which he described children suffering from spasticity and stiffness of extremities, deformities of upper and lower extremities, paresis and paralysis [3, 4]. Little compared the stiffness that his patients had to tetanus spasms. The connection between the above disorders and perinatal disorders became more and more clear to him [4]. As his experience grew, Little classified children’s disorders into groups [3]. Of particular importance was the connection he made between bone, joint and muscle deformities and disorders of the neurological system. He associated these disorders with prematurity, difficult delivery, in particular forceps delivery, as well as perinatal asphyxia and tremors. Little was the first person to explain in detail the mechanism involved in muscle contractures, particularly spastic ones [3]. Additionally, Little described cases of pseudohypertrophic muscular dystrophy [3] at around the same time as Edward Meryon and before the eponymous description of Guillaume Duchenne [5]. As a result of his observations and growing experience, Little became more and more aware that surgical treatment of cerebral palsy had its limitations. The initial fascination with tenotomies gave way to a much more cautious approach. This was caused by Little’s observations that some treatment, corrective in theory, in fact impaired children’s motor function. This was an exceptionally mature idea at the time when orthopaedics was going through the period of “post-operative disaster” [6]. This knowledge was reflected in his major work on this topic [7] based on the observations of over 200 patients. Little presented his main ideas in 1861 in a meeting of the Obstetrical Society of London. His views stirred a heated debate. He highlighted that the disease was caused by problems during pregnancy and delivery. He underlined the impact of such conditions as placenta praevia and prematurity. Little believed that cerebral palsy resulted from post-partum asphyxia, which distorted the blood flow and in this way damaged the child’s brain. He was convinced that the cause of the disease was lack of oxygen during delivery, while rejecting the hypothesis of the impact of “injuries” suffered during delivery. Little also described in detail different types of paralysis: hemiplegia, diplegia (sometimes known as “Little’s diplegia”), and tetraplegia. [7]. He also pointed to the possibility of flaccid paralysis. He saw the link between a degree of paresis and a degree of mental retardation in patients [7].

Little was, therefore, one of the first to describe what might now be called “cerebral palsy”, although he never used this term in any of his works. The first person to do so was William Osler in 1888 [8]. Sigmund Freud (1856–1939) described changes to the brain, linked them with types of paresis and refined the concept of spastic diplegia [9].

In 1893, Little resigned from his post at the London Hospital, but he still practiced as a surgeon and continued his research. He retired because of his progressing deafness and moved to Ryarsh in Kent, where he died on 7 July 1894.

William Little married Eliza Templin. They had 11 children, 7 survived into adulthood. Two of his sons continued his orthopaedic mission, and in 1918 one of them, Ernest Muirhead Little (1854–1935), became the first President of the British Orthopaedic Association.

The techniques used today in the surgery of muscular dystrophy were originated by Stromeyer and Little. William Little was one of the first to work in the field between neurology and orthopaedics and his important work continues to have influence on both these fields.
